# Emerging pharmacotherapy for cancer patients with cognitive dysfunction

**DOI:** 10.1186/1471-2377-13-153

**Published:** 2013-10-24

**Authors:** Justin Davis, Fiona M Ahlberg, Michael Berk, David M Ashley, Mustafa Khasraw

**Affiliations:** 1 School of Medicine of Deakin University, Geelong, VIC, Australia; 2 Andrew Love Cancer Centre, Geelong Hospital, Geelong, VIC, Australia; 3 Department of Psychiatry, Orygen Youth Health and the Florey Institute for Neuroscience and Mental Health University of Melbourne, Parkville, VIC, Australia; 4 Royal Melbourne Hospitals, Parkville, VIC, Australia; 5 Deakin University, Andrew Love Cancer Centre, 70 Swanston St, Geelong, Vic 3220, Australia

**Keywords:** Cancer, Cognitive impairment, Chemotherapy, Cognitive dysfunction, Pharmacotherapy

## Abstract

Advances in the diagnosis and multi-modality treatment of cancer have increased survival rates for many cancer types leading to an increasing load of long-term sequelae of therapy, including that of cognitive dysfunction. The cytotoxic nature of chemotherapeutic agents may also reduce neurogenesis, a key component of the physiology of memory and cognition, with ramifications for the patient’s mood and other cognition disorders. Similarly radiotherapy employed as a therapeutic or prophylactic tool in the treatment of primary or metastatic disease may significantly affect cognition. A number of emerging pharmacotherapies are under investigation for the treatment of cognitive dysfunction experienced by cancer patients. Recent data from clinical trials is reviewed involving the stimulants modafinil and methylphenidate, mood stabiliser lithium, anti-Alzheimer’s drugs memantine and donepezil, as well as other agents which are currently being explored within dementia, animal, and cell culture models to evaluate their use in treating cognitive dysfunction.

## Introduction

### Cognitive dysfunction in cancer patients

The possibility that chemotherapy could induce cognitive changes became a focus of research after breast cancer patients began to complain of a vague mental 'fogginess’ that developed during their treatment. This later came to be defined with the unfortunate term 'chemobrain’ [1-7]. The word chemobrain is now used to describe a constellation of symptoms across a variety of cognitive domains, with the most commonly reported problems being in areas of short-term memory, concentration and attention, and planning [4]. The term chemobrain is somewhat of a misnomer, as cognitive complaints may be seen in a subset of patients *prior* to receiving any treatment [1-6,8]. It has become increasingly clear that this pre-treatment dysfunction may be encountered across different tumour types.

The cognitive domains which are most affected by chemotherapy or the cancer itself vary according to study design and testing method. With regards to patients with breast cancer Wefel *et al.* found processing speed, attention, nonverbal memory, executive function, and problem solving to be the most vulnerable to the effects of chemotherapy [5,9]. Correa and Hess (2012) similarly documented deficiencies in executive function, memory, and reaction time in patients with ovarian cancer [10]. Patients who have been treated for primary brain tumours also display a similar set of cognitive defects to those aforementioned during long term follow up, with decreased attention, difficulty with expressive language, executive function, processing speed, and poor short-term memory seen among survivors of primary brain tumours [11,12]. Within the paediatric population it is generally accepted that underlying defects in attention and/or working memory following treatment for acute lymphoblastic leukaemia (ALL) or childhood brain tumours mediate the observed declines in intelligence quotient (IQ) and academic achievement seen during longitudinal follow-up [13,14]. The deficiencies seen mirror those found in children with attention deficit hyperactivity disorder (ADHD), particularly those symptoms of the primary inattentive type of ADHD [15]. This association has led researchers to trial methylphenidate in children who display these cognitive defects after treatment to determine whether a similar response profile could be obtained [15,16]. This and other trials involving methylphenidate will be discussed later in this review.

Largely due to variations in study and testing design and differing definitions of impairment, there is a large variation in reported rates of cognitive impairment following treatment. Reported figures range from 15-90% of patients, and even higher figures have been reported from patients with brain tumours [7,9,17].

There are challenges in unravelling the cause of cognitive dysfunction in patients with cancer. The changes caused can often be confused or confounded by other issues seen in cancer treatment including anxiety, depression, sleep disturbance, or fatigue [1,7,18]. Increased levels of circulating cytokines have been implicated in mediating some of these changes, especially so in the case of pre-treatment cognitive dysfunction [19].

Defining exactly what changes chemotherapy induces has remained elusive. One proposed mechanism is chemotherapy-induced de-oxyribonucleic acid (DNA) damage, either directly or indirectly through oxidative stress. Chemotherapy may also induce hormonal changes, an immunologic-driven process, microvascular injury, and/or decreased neurogenesis [9,20].

Cranial irradiation can also induce pathological changes that have been described as similar to the disease process in Alzheimer’s Disease [7,21]. Radiation-induced neurocognitive changes are often seen early in the post-radiotherapy period, and more subtle changes may also be observed several years later in patients who are long-term survivors [22].

Glucocorticoids, the use of which is highly prevalent in patients with cancer, have been associated with a 5–50% incidence of glucocorticoid-induced psychiatric syndromes including mania, insomnia, restlessness, euphoria, and increased motor activity [5]. The use of opioids can produce drowsiness and decreased cognitive function [23]. In the late stages of treatment and particularly in the palliative care setting, opioid-induced delirium can be observed, however, delirium in this setting is often multi-factorial [7]. It is well known that methotrexate and 5-fluorouracil are directly neurotoxic and can cause diffuse white matter changes, and agents such as doxorubicin**,** paclitaxel, cisplatin, and cyclophosphamide are also implicated [9].

Factors that increase one’s risk of developing neurotoxicity related to chemotherapy include: the use of high-dose regimens or exposure to high doses of the parent drug or its metabolite due to impaired systemic clearance or pharmacologic modification of hepatic enzyme systems; synergistic effects of different chemotherapeutic agents; the synergistic effect of multi-modality treatment, which includes radiotherapy; agents that can cross or processes that disrupt the blood–brain barrier (BBB); and intrathecal administration of cytotoxic chemicals [9].

Dysfunction induced by therapy also appears to be related to the intensity of therapy. Studies performed in patients with breast cancer have shown that the relative risk of cognitive dysfunction is 3·5 times higher in patients who received intensive regimes of chemotherapy (cyclophosphamide, high-dose thiotepa-carboplatin) compared to patients who were treated with cyclophosphamide alone [24].

Significant numbers of cancer patients suffer cognitive impairments and concurrent mood disorders which may impact on their functional ability. This may be due to cancer within the Central Nervous System (CNS), paraneoplastic effects and the various treatments modalities offered. This paper offers a review of the emerging pharmacotherapies, which attempt to prevent or minimise cognitive dysfunction in these settings.

## Review

### Pharmacotherapy for cancer patients with cognitive dysfunction

#### ***Modafinil***

Modafinil is a novel stimulant that currently has United States (US) Food and Drug Administration (FDA) authority for use in narcolepsy [25]. The exact mechanism of action for modafinil remains largely unknown. It has been demonstrated that in a similar fashion to derivatives of amphetamine modafinil is able to increase catecholaminergic signalling. However in contrast to the amphetamine derivatives, which act broadly throughout the cortex and striatum to produce excitation, modafinil has been shown to act primarily within sleep-wake centres in the anterior hypothalamus to promote wakefulness [26,27]. It is believed to do this through inhibition of gamma-amino butyric acid (GABA) outflow from the sleep-generating ventrolateral pre-optic area of the hypothalamus [28]. Modafinil has not been shown to stimulate dopaminergic transmission throughout the cortex or produce significant sympathomimetic side effects [28]. This selective action on thalamic sleep-wake centres is thought to be the reason that modafinil displays a lower side effect profile as compared to methylphenidate in addition to having a lower abuse potential [26-28].

Whilst many clinical trials have looked at the effects of modafinil on fatigue within cancer patients, there have been comparatively few which have looked primarily at cognitive dysfunction. Kohli *et al.* (2009) looked at the effect of modafinil on cognitive dysfunction as a secondary analysis of a previous trial which looked at its efficacy in the treatment of cancer related fatigue. In an open-label Phase I/ II study, 82 women with an initial diagnosis of breast cancer who had been treated with chemotherapy and/or radiation therapy one month prior to the study were assessed. Patients had previously been enrolled within a primary study conducted by the authors which looked at the effects of modafinil on self-reported fatigue on the basis of a score of greater than two on the Brief Fatigue Inventory (BFI) [29] and only those patients who had given a score of 2 (indicating worst fatigue) were invited to participate. In Phase I of the current study, patients received 200mg of modafinil once daily for four weeks, and those who had favourable responses were then randomised to modafinil or placebo for an additional four weeks in Phase II. Cognitive Drug Research (CDR) computerised testing was used as a battery at baseline. After completion of both phases, modafinil was found to demonstrate significant improvement in speed of memory and episodic memory within the open label phase I, and those who were assigned modafinil within the placebo controlled phase II continued to show improvement in those domains when compared to women who were randomised to receive placebo. As such the authors concluded that modafinil might be useful in enhancing memory and attention skills.

Lundorff *et al.* (2009) in a double blind, randomised cross over trial looked at the effect of modafinil against placebo on cognitive function as a primary outcome with secondary aims of looking at the effectiveness of modafinil on other symptoms. 28 patients with advanced cancer in a palliative care setting who had a self-reported tiredness score of five or more (range 0–10, where 0 was no tiredness and 10 was the worst possible tiredness) and a Karnofsky Performance Status of 40–70 were invited to participate. On day one patients randomly received 200 mg of modafinil or placebo before being switched to the alternative treatment on day four [30]. The Trial Making Tests A and B, Finger Tapping Test (FTT), and Edmonton Symptom Assessment System (ESAS) were performed at baseline and then four hours after the tablets were taken. The authors showed a statistical improvement on modafinil in all three tests of cognitive function compared to placebo and additionally reported that subjective scores of depression and drowsiness were improved in those who had been treated with modafinil.

Blackhall *et al.* (2009) performed a pilot study which looked primarily at the effect of modafinil on cancer-related fatigue and secondarily on outcomes of cognitive function [31]. 27 patients with a variety of different cancers were recruited. Modafinil was given at 100 mg daily for two weeks, then 200 mg for a further two weeks. Assessment of cognitive function in the study was done using the Hopkins Verbal Learning Test (HVLT), the Grooved Pegboard Test, the Controlled Oral Word Association Test (COWAT) and the Trail Making Test A was performed at baseline, after two weeks and after four weeks of treatment with modafinil. The authors found that in comparison to the positive effects on fatigue and self-reported quality of life in their primary outcomes, modafinil was not associated with significant improvement in measures of cognitive function.

#### ***Methylphenidate***

Methylphenidate has been far more extensively studied in relation to cognitive dysfunction amongst cancer patients that the other stimulants reviewed here, particularly in the paediatric population. Methylphenidate acts in a similar fashion to amphetamines. It is a dopaminergic and noradrenergic agonist that increases concentrations of these neurotransmitters in the fronto-striatal, network (the network which governs attention) [32,33]. It also reduces dopamine uptake at synapses and has been shown to inhibit the action of monoamine oxidase [33]. Owing to the ability of methylphenidate to induce widespread cortical activation it is also associated with more significant side effects than modafinil. The most troublesome of methylphenidate’s side effects include; appetite suppression, anxiety, restlessness, sweating, hallucinations, tolerance, and addictive properties [34].

Mar Fan *et al.* (2008) in a randomised, placebo-controlled double-blind trial studied the effects of methylphenidate on both cognitive function and fatigue in women undergoing adjuvant chemotherapy for fully resected early breast cancer. The authors looked at 57 women who initially randomised to 5 mg twice daily of placebo run in [35]. Those patients who were compliant with medication and the testing battery were then randomised to either 5 mg of methylphenidate twice daily or placebo. Patients were assessed at baseline, at the end of their chemotherapy cycles, and after six months with the Mini-Mental State Examination (MMSE), High Sensitivity Cognitive Screen (HSCS) and the Hopkins Verbal Learning Test-Revised (HVLT-R). The authors found no statistically significant difference in cognitive function between methylphenidate or placebo on HVLT-R or HSCS scores at the conclusion of their trial.

Lower *et al.* (2009) in a randomised, placebo-controlled double-blind study looked at the effects of methylphenidate primarily on fatigue with secondary measures of cognitive function [36]. One hundred and fifty-four patients with predominantly breast or ovarian cancer who had previously completed initial cycles of treatment greater than 2 months before study initiation. Measures of cognitive function were done with the HSCS. Whilst the authors found no significant improvement in cognitive function as measured by the HSCS at the conclusion of the study period, they noted that their study was not powered to fully assess such a secondary endpoint and regarded such an outcome as exploratory only.

Gagnon *et al.* (2005) performed a prospective clinical study which looked at the efficacy of methylphenidate in cancer patients who were also suffering from hypoactive delirium. A total of 14 patients with hypoactive delirium were included in the study. Patients were given 10mg of methylphenidate twice daily and were asked to perform an MMSE one hour after the administration of methylphenidate on each follow-up visit, daily for inpatients, every three to four days for outpatients [23]. Improvement from the baseline MMSE score of 21 out of 30 was noted in all patients following treatment, with the median score improving to 28. The authors concluded that the use of methylphenidate was the reason for their patients’ rather surprising improvement in MMSE scores.

Gehrig *et al.* (2012) performed a small, open-label randomised pilot trial which directly compared both immediate release and long acting forms of methylphenidate against modafinil. A total of 24 patients with the diagnosis of a primary brain tumour who subjectively complained of cognitive dysfunction were recruited into the trial [37]. Patients were asked to complete several psychometric tests which included; HVLT-R, COWAT, trail making tests A and B, and the Abbreviated Wechsler Intelligence Scale for Children (WAIS-III) digit span and digit symbol tests at baseline and again at four weeks. However due to slow accrual the trial was terminated early. Whilst there was suggestion of beneficial effects on patient-reported measures for both drugs, the authors advise caution in interpreting the results due to the small study size.

Methylphenidate has also been studied within paediatric populations who experience cognitive dysfunction, particularly attention and learning disorders following treatment for childhood cancers. Conklin *et al.* (2007) performed a double-blind cross over trial looking at 122 children who were survivors of either acute lymphoblastic leukaemia or primary brain tumours. Such patients had completed treatment regimes a minimum of 12 months prior and were between 6 and 18 years of age who currently had learning impairments thought to be a consequence of their treatment regimes [14]. Participants received either methylphenidate 0.60 mg/kg to a maximum of 20 mg or placebo and were subsequently switched to the alternative treatment the following day. 90 minutes after administration participants were asked to complete; the Brief Continuous Performance Test (CPT), the California Verbal Learning Test Children’s Version (CVLT-C), the Visual-Auditory Learning Test (VAL), the Wide Range Achievement Test (WRAT) and Barkley’s Side Effects Rating Scales. The authors found that the domains which showed the most benefit in those who received methylphenidate were those which looked at measures of processing speed and response tendency. Measures of attention did not significantly improve, nor did those which looked at memory encoding/retrieval or productivity.

#### ***Donepezil***

Donepezil is a centrally acting anti-cholinergic that is that is currently used mainly in the treatment of Alzheimer’s Disease [38]. The major side effects of donepezil relate to its anti-cholinergic properties, and include; vomiting, diarrhoea, and weight loss [11,38]. As hippocampal-related memory loss is seen as a common side effect of chemotherapy, it is reasonable to assume that donepezil might have beneficial effects on cognitive function when used in these patients [39,40].

Winocur *et al.* (2011) looked at the effects of donepezil on cognitive impairment in a murine model where cognitive dysfunction was induced with a combination of methotrexate and 5-fluorouracil [40]. Four groups of mice were given: chemotherapy only, chemotherapy and donepezil, saline only, or saline and donepezil and then subjected to learning and memory tests within a water maze. The tests included; a standard spatial memory test, non-spatial tests of cued memory and tests of non-matching to sample rule learning and delayed non-matching to sample. It was found that chemotherapy-induced cognitive dysfunction was significantly reduced in the group treated with chemotherapy and donepezil a finding that was so strong their performance results were similar to the group who had received saline only. The authors suggested that their study showed that chemotherapy did induce chronic cognitive changes and that donepezil could be used to ameliorate some of these changes.

A number of relatively small studies have looked at the efficacy of donepezil in cancer patients. Castellino *et al.* (2012) performed a pilot study of donepezil in 11 children who were survivors of primary brain tumours who had received greater than or equal to 23·5Gy during the course of their treatment [11]. Such patients had to have not received treatment in the past 12 months. The study was conducted as an open label trial with escalation to the target dose of donepezil by week 6 followed by a washout period of the drug from weeks 24–36. Neurocognitive assessment was conducted at baseline and at weeks 12, 24 and 36 following washout. Assessment was performed with the Dellis-Kaplan Executive Function (D-KEF), Wide Range Assessment of Memory and Learning 2^nd^ edition (WRAML-2), CPT, the Wechsler Intelligence Scale for Children (WISC-IV) and Woodcock Reading Mastery Test and III Calculations. The authors found preliminary evidence for the efficacy of donepezil in the domains of executive function and memory, with significant performance improvements noted within the D-KEFs tower task.

Shaw *et al.* (2006) performed a prospective, open-label Phase II study of donepezil in 34 irradiated, mostly low-grade glioma patients. Such patients had completed treatment courses 6 months prior to study enrolment with a Karnofsky Performance Status > 70. Donepezil was given at 5 mg/d for six weeks, then 10 mg/d for 18 weeks, followed by a 6-week washout period. The cognitive test battery employed the use of the MMSE, Trail Making Test A and Digit Span Test, the Revised Ray-Osterrieth Complex Figure Test, Controlled Oral Word Association Test, CVLT and Trail Making Test B at baseline and then weeks 6, 12, 24 and 30. Scores significantly improved between baseline and week 24 on measures of attention/concentration, verbal memory and figural memory. A tend for improvement in verbal fluency was also noted [12].

#### ***Lithium***

Lithium, a cation that is mainly used in the management of bipolar mood disorder, is another agent that has been shown to have both neuroprotective and anti-apoptotic effects [41]. Epidemiologic studies in patients on long-term lithium for bipolar disorder suggest that they are less likely to have cognitive impairment when compared with similar patients managed on mood stabilisers other than lithium [42]. Yazlovitskaya *et al.* looked at the neuro-protective effects of lithium against cranial irradiation in a murine model. Mice were pre-treated with intraperitoneal lithium at 40 or 80 mg/kg for seven days whilst a control group was given saline at 150 mmol/L. The animals were then either irradiated with 1–10 Gy of radiation and sacrificed 10 hours later for histologic staining, or irradiated with 7 Gy and tested on the Morris Water Maze 6 weeks later. The authors found that the pre-treatment of mice with lithium for seven days before cranial irradiation confers a significant anti-apoptotic effect, particularly within the hippocampus where control mice experienced severely altered hippocampal function. Such findings have been echoed by other researchers who have also looked into the potential protective effects of lithium. Inouye *et al.* (1995) pre-treated mice with 10 micromol/g of lithium 2 hours before irradiation with 0.5 Gy and found that the histologic manifestations were delayed in mice who had been pre-treated with lithium [43]. Cimarosti *et al.,* looking at the effects of ischaemia in sections of rat hippocampi found that the use of lithium in this situation conferred a neuroprotective effect against the ischaemic state [44]. There has been one early-phase study, presented in abstract form, in which lithium was used as a neuroprotectant [45,46]. Another pilot study using lithium as a neuroprotectant in Small Cell Lung Cancer Patients receiving prophylactic cranial irradiation is ongoing. Efficacy data from a larger study in patients with brain metastases treated with whole brain radiotherapy are in planning.

#### ***Peroxisome proliferator-activated receptor agonists***

Peroxisome proliferator-activated receptors (PPARs) are members of the nuclearhormone receptor superfamily. Rosiglitazone and Pioglitazone, also called thiazolidinedione or glitazones, are used as oral hypoglycemic agents or insulin sensitisers for the treatment of diabetes. Glitazones act by activating PPARs with greatest specificity for the PPARγ (*gamma*) member of the superfamily. Administering pioglitazone to young adult male rats starting three days prior to, during, and for four or 54 weeks after the completion of a total 40 Gy dose of whole brain radiation prevented radiation-induced cognitive dysfunction assessed 52 weeks after the radiation delivery [47]. An early-phase clinical trial has been initiated to determine the dose of pioglitazone that can be given safely in this setting.

#### ***Angiotensin receptor blockers***

Other drugs that have been investigated in animal models include Angiotensin Receptor Blockers (ARB), commonly used as antihypertensives, as well as the chronic administration of angiotensin type 1 (AT1) receptor antagonist, L-158,809. The latter, when administered for three days in rats before, during, and only five weeks after radiotherapy, prevented cognitive dysfunction observed 26 weeks post-irradiation [48].

#### ***Targeting the nitric oxide pathway***

Nitric oxide synthase (NOS) cofactor 5,6,7,8-tetrahydrobiopterin (BH4) has also been proposed as an agent to reduce radiation-induced cognitive damage. This is based on considering BH4 as an essential cofactor for NOS enzymes. Insufficient BH4 may lead to uncoupling of the NOS enzyme. In an uncoupled state, NOS can produce highly oxidative radicals at the cost of NO. Under conditions of oxidative stress such as after radiation exposure, BH4 availability might be reduced because of rapid oxidation. As a result, free radical-induced BH4 insufficiency may increase the oxidative burden and hinder the NO-dependent endothelial function [49].

#### ***Memantine***

Recently, Brown et al. (2012) reported results of a randomised, placebo-controlled, double-blind trial looked at the effects of memantine. This agent is a N-methyl D-aspartate (NMDA) antagonist which has found prior use in the treatment of Alzheimer’s Disease, in patients who had received whole brain radiation therapy for metastatic disease. A total of 508 eligible patients were randomised to either receive placebo or 20 mg of memantine within 3 days of the initiation of radiotherapy for 24 weeks. Participants were assessed using the HVLT-R delayed recall, COWAT, and Trail Making Test 1 testing battery at baseline and weeks 8.16, 24 and 52. The authors showed that there was a significantly longer time to cognitive decline in those who had been randomised to receive memantine. In addition, fewer patients who were on memantine experienced decline in COWAT or the Trail Making Test A compared to placebo [50].

#### ***Other potential neuroprotective agents***

Whilst few of the following agents have been studied for their beneficial effects in the management of cognitive dysfunction within cancer patients, research into their efficacy is an area of growing research within the fields of mood disorders and dementing illnesses. As such they are presented with those limitations in mind with the thought that they may one day be further researched specifically within a population of cancer and chemotherapy induced cognitive impairment.

#### ***N-acetyl cysteine***

N-acetyl cysteine (NAC) functions as a readily bio-available source of cysteine, the rate limiting step in the synthesis of the potent antioxidant glutathione and a precursor to glutathione, which has been shown to be one of the most important scavengers of free radicals within neural tissue [51,52]. It has also been shown to blunt the inflammatory response within the brain within a number of conditions, including; multiple sclerosis, endotoxaemia, ischaemia-reperfusion injury, and hypoxic ischaemic encephalopathy [53]. In an underpowered placebo-controlled study in bipolar disorder, no effect on cognition was noted [personal communication].

While NAC has not been subject to studies within cancer patients with cognitive dysfunction, it has been trialled in dementia patients (specifically in those with Alzheimer’s Disease) and in patients with bipolar mood disorder [51,52]. In a randomised, double-blind trial of NAC against placebo in 43 patients who met the diagnostic criteria for probable Alzheimer’s Disease, Adair *et al.* gave NAC at a dose of 50 mg/kg/d and measured outcomes using the MMSE at baseline, 12 weeks and 24 weeks. The authors found that active treatment with NAC failed to significantly alter primary outcome measures, but did note that a direct comparison of interval change favoured the NAC group for most outcome measures although only a few of these were significantly different [54].

#### ***Resveratrol***

Interest in resveratrol, a polyphenolic phytoalexin found within plant sources such as berries, nuts and grapes, was first aroused by epidemiologic studies that suggested that subjects who consumed a high proportion of wine had a lesser incidence of dementia than populations who did not [55]. It has been suggested that the moderate consumption of red wine, rich in compounds such as resveratrol, helps to ameliorate some of the detrimental cognitive effects seen in dementing illnesses such as Alzheimer’s Disease [56,57]. The findings of these studies have been reinforced by mouse models in which resveratrol demonstrated anti-apoptotic, antioxidant and acetylcholinesterase activity controlling properties [56]. While resveratrol has reached Phase II clinical trials [57], the applicability of using it within cancer patients remains to be established. The use of resveratrol and related compounds has certainly been questioned in trials of patients with Alzheimer’s Disease with randomised trials showing no cognitive benefit of these drugs. It should be noted that these trials were of a short duration (<2 years) and may not have had enough power to detect a statistically significant difference [57]. Another hypothesis is that the study of resveratrol and related antioxidants such as curcumin or catechins have been almost exclusively performed in animal models or cell cultures that show little of the extensive neuronal loss that is seen in patients with advanced dementia [58]. Therefore, the potential benefits of antioxidants such as resveratrol remain the subject of ongoing research.

#### ***Non-steroidal anti-inflammatory drugs***

Non-Steroidal Anti-Inflammatory Drugs (NSAIDS) fall under the same curious category as resveratrol and related antioxidants in that, while benefits have been shown in preventing cognitive decline in epidemiological and cell culture studies, these benefits have failed to translate into meaningful clinical results in controlled trials. The suggestion that NSAIDs might be beneficial in halting the rate of cognitive decline came from epidemiological studies of rheumatology patients taking regular NSAIDs which again showed a lower incidence of dementia than other populations [57]. Community-based prospective studies and some cross-sectional ones also suggested a benefit to regular aspirin use and the preservation of global cognitive performance and episodic memory [59]. Multiple pre-clinical studies in transgenic mice showed very favourable results in cognitive protection, which suggested a rather straightforward relationship between the induction of cyclooxygenase (COX) activity and subsequent prostaglandin-induced memory dysfunction [60]. However the relationship between inflammation and cognitive decline proved to be far more complex than what was originally suggested, as evidenced by randomised, placebo-controlled trials that showed no benefit from the traditional NSAIDs nor from the newer selective COX-2 inhibitors [61].

Among the NSAIDs, aspirin has received particularly heavy attention due to its already proven role in the prevention of cardiovascular disease. Aspirin has been suggested to slightly reduce the incidence of mortality from cancer, as indicated by long-term observational studies [62]. Kang *et al.* in a cohort study using data from the women’s health study, a randomised, placebo-controlled, double-blind trial with nearly ten years of treatment in over 6,000 women failed to show any benefit of low-dose aspirin, with similar cognitive performance after 5.6 years of treatment, 9.6 years and 3–6 years of subsequent follow up compared to those who had received a placebo during that time [63]. A recent *Cochrane* review on the use of NSAIDs in slowing the cognitive decline seen in Alzheimer’s Disease found no significant improvement in decline in cognitive function among patients on aspirin, traditional NSAIDs, selective COX-2 inhibitors, or glucocorticoids, and could not recommend their routine use in Alzheimer’s Disease [64].

## Conclusion

While gains in the understanding of the pathogenesis of cancer-induced cognitive dysfunction has been made in the 20 years since breast cancer patients first began reporting the symptoms of 'chemobrain’, the process of determining how best to manage it remains a significant challenge. There is a growing body of evidence that the use of stimulants such as modafinil or methylphenidate can help to improve cognitive measures in cancer patients with chemotherapy induced cognitive dysfunction (Table 1). It would appear that modafinil is currently favoured over methylphenidate because of its lesser side effect profile and the associated stigma which methylphenidate carries with it. The acetylcholinesterase inhibitor donepezil has also received attention due to its role in slowing the cognitive decline seen in Alzheimer’s Disease. However the results of using donepezil in cancer patients with cognitive dysfunction have been less encouraging. The question of whether these agents can provide any definite improvement in the lives of cancer patients will require larger and more substantially powered trials before it can be fully answered.

**Table 1:** A summary of the evidence regarding the potential benefit of pharmacologic intervention in cancer patients with cognitive impairment and fatigue

**Medication**	**Mechanism of action**	**Evidence of impact on cognition in cancer patients**
MODAFINIL	Thought to inhibit GABA outflow tracts within the ventro-lateral preoptic area of the hypothalamus.	Clinical trials have looked at fatigue as a primary outcome and cognition as a secondary one, although most have demonstrated efficacy in improving cognition.
METHYLPHENIDATE	Dopaminergic and noradrenergic agonist which acts to increase levels of these neurotransmitters within the frontal striatal network.	Randomised, double-blind trials in childhood cancer patients suggest efficacy, but no evidence of superiority over placebo in adult trials.
DONEPEZIL	Centrally acting anticholinergic used in the management of Alzheimer’s Disease.	Open-label Phase II studies in glioma patients suggested statistically significant improvement in cognitive functioning.
LITHIUM	Cation with an unknown mechanism of action used in the management of bipolar mood disorder.	Murine models and efficacy data from bipolar patients suggest anti-apoptotic and neuroprotective potential.
PPARs	Nuclear hormone receptor functioning as a transcription factor, targeted in the control of type II diabetes mellitus.	Murine models have demonstrated protection against radiation induced cognitive dysfunction.
ARBs	Antagonism of the angiotensin II receptor used in the management of hypertension.	Murine models have demonstrated protection against radiation induced cognitive dysfunction.
NOS	Produces NO which functions as a neurotransmitter.	None, theoretical benefit through induction of NOS and subsequent antioxidant activities.
MEMANTINE	NMDA antagonist used in the management of Alzheimer’s Disease.	Recent randomised placebo-controlled double-blind trial in patients receiving whole brain radiation showed longer time to cognitive decline over placebo.
N-ACETYL CYSTEINE	Provides cysteine, the rate limiting step in the synthesis of the anti-oxidant glutathione.	Limited evidence of efficacy in Phase II trials in Alzheimer’s patients.
RESVERATROL	Unknown, demonstrates anti-apoptotic and anti-oxidant properties within cell cultures.	Phase II studies in Alzheimer’s patients suggest little to no cognitive benefit.
NSAIDS	Inhibition of COX isoforms.	Recent Cochrane review indicates no significant impact in slowing cognitive decline in Alzheimer’s patients, Women’s Health study subanalysis echoes this finding.

A host of other agents are being studied in relation to other neurocognitive diseases such as dementia, but these again remain to be fully evaluated in cancer patients. The role of NSAIDs in preventing cognitive decline is being questioned due to poor performance in randomised controlled trials. Other compounds, including N-acetylcysteine, resveratrol, and lithium, have shown good initial performance in cell culture, animal models, and some pilot studies, but remain to be fully explored in appropriately designed clinical trials.

The role of neuroprotective agents in cancer-induced cognitive dysfunction is becoming clearer but still remains to be fully defined. As more is learned about the processes that contribute to cognitive decline in these patients further opportunities to target these changes with neuroprotective agents will become available. However the compounds currently being explored within the literature need to be further evaluated before concrete clinical recommendations can be made.

## Abbreviations

ALL: Acute lymphoblastic leukaemia; IQ: Intelligence quotient; ADHD: Attention deficit hyperactivity Disorder; DNA: De-oxyribonucleic acid; BBB: Blood–brain barrier; CNS: Central nervous system; US: United states; FDA: Food and drug administration; GABA: Gamma-amino butyric acid; BFI: Brief fatigue inventory; CDR: Cognitive drug research; FTT: Finger tapping test; ESAS: Edmonton symptom assessment System; HVLT: Hopkins verbal learning test; COWAT: Controlled oral word association test; MMSE: Mini-mental state examination; HSCS: High sensitivity cognitive screen; HVLT-R: Hopkins verbal learning test-revised; WAIS-III: Abbreviated wechsler intelligence scale for children; CPT: Brief continuous performance test; CVLT-C: California verbal learning test children’s version; VAL: Visual-auditory learning test; WRAT: Wide range achievement test; D-KEF: Dellis-Kaplan executive function; WRAML-2: Wide range assessment of memory and learning 2^nd^ edition; WISC-IV: Wechsler intelligence scale for children; PPARs: Peroxisome proliferator-activated receptors; ARB: Angiotensin receptor blockers; NOS: Nitric oxide synthase; BH4: 5,6,7,8-tetrahydrobiopterin; NMDA: N-methyl D-aspartate; NAC: N-acetyl cysteine; NSAIDS: Non-steroidal anti-inflammatory Drugs; COX: Cyclooxygenase.

## Competing interest

The authors declare that they have no conflicts of interest.

## Authors’ contribution

MK drafted the structure of the manuscript. FMA and JD performed the literature search. MK, FMA and JD selected the references, assessed the quality of that evidence and initiated the writing. All authors contributed to the writing and approval of the final manuscript.

## Pre-publication history

The pre-publication history for this paper can be accessed here:

http://www.biomedcentral.com/1471-2377/13/153/prepub
